# Set-Based Rare Variant Expression Quantitative Trait Loci in Blood and Brain from Alzheimer Disease Study Participants

**DOI:** 10.3390/genes12030419

**Published:** 2021-03-15

**Authors:** Devanshi Patel, Xiaoling Zhang, John J. Farrell, Kathryn L. Lunetta, Lindsay A. Farrer

**Affiliations:** 1 Bioinformatics Graduate Program, Boston University, Boston, MA 02215, USA; dpatel@bu.edu; 2 Department of Medicine (Biomedical Genetics), Boston University School of Medicine, Boston, MA 02118, USA; zhangxl@bu.edu (X.Z.); farrell@bu.edu (J.J.F.); 3 Department of Biostatistics, Boston University School of Public Health, Boston, MA 02118, USA; klunetta@bu.edu; 4 Department of Ophthalmology, Boston University School of Medicine, Boston, MA 02118, USA; 5 Department of Neurology, Boston University School of Medicine, Boston, MA 02118, USA; 6 Department of Epidemiology, Boston University School of Public Health, Boston, MA 02118, USA

**Keywords:** Alzheimer disease, expression quantitative trait loci (eQTL), rare variants, set-based eQTL, SKAT-O, pathways, immune system, inflammation, ROSMAP, ADNI

## Abstract

Because studies of rare variant effects on gene expression have limited power, we investigated set-based methods to identify rare expression quantitative trait loci (eQTL) related to Alzheimer disease (AD). Gene-level and pathway-level cis rare-eQTL mapping was performed genome-wide using gene expression data derived from blood donated by 713 Alzheimer’s Disease Neuroimaging Initiative participants and from brain tissues donated by 475 Religious Orders Study/Memory and Aging Project participants. The association of gene or pathway expression with a set of all cis potentially regulatory low-frequency and rare variants within 1 Mb of genes was evaluated using SKAT-O. A total of 65 genes expressed in the brain were significant targets for rare expression single nucleotide polymorphisms (eSNPs) among which 17% (11/65) included established AD genes *HLA-DRB1* and *HLA-DRB5*. In the blood, 307 genes were significant targets for rare eSNPs. In the blood and the brain, *GNMT*, *LDHC*, *RBPMS2*, *DUS2*, and *HP* were targets for significant eSNPs. Pathway enrichment analysis revealed significant pathways in the brain (*n* = 9) and blood (*n* = 16). Pathways for apoptosis signaling, cholecystokinin receptor (CCKR) signaling, and inflammation mediated by chemokine and cytokine signaling were common to both tissues. Significant rare eQTLs in inflammation pathways included five genes in the blood (*ALOX5AP*, *CXCR2*, *FPR2*, *GRB2*, *IFNAR1*) that were previously linked to AD. This study identified several significant gene- and pathway-level rare eQTLs, which further confirmed the importance of the immune system and inflammation in AD and highlighted the advantages of using a set-based eQTL approach for evaluating the effect of low-frequency and rare variants on gene expression.

## 1. Introduction

Late-onset Alzheimer disease (AD) is the most common type of dementia that affects an estimated 5.7 million individuals aged 65 years and older in the United States, with the number projected to rise to 14 million by 2050 [1]. AD is highly heritable (h2 = 58–79%) [2], but common variants explain only one-third of the genetic portion of AD risk [2]. Highly penetrant rare variants may account for some of the missing heritability [3]. Whole-exome sequencing studies have identified robust AD associations with rare missense variants in *TREM2, AKAP9, UNC5C, ZNF655, IGHG3, CASP7* and *NOTCH3* [4,5,6,7,8,9], and it is expected that more AD-related rare variants will be identified by whole-genome sequencing (WGS) studies, because some rare variants, including those in non-coding regions, likely contribute to AD risk. However, identification of genes that are impacted by these rare variants, and thus likely have a functional role in AD, remains challenging.

Some AD risk variants are associated with gene expression, as demonstrated by recent expression quantitative trait locus (eQTL) studies [10,11]. Rare variants may contribute to extreme gene expression within a single tissue or across multiple tissues [12,13,14,15]. However, genome-wide studies of rare eQTLs are generally underpowered to obtain significant results. Although gene-based tests, which test the aggregate effects of multiple variants, are commonly used to evaluate the association of a disease with rare variants, only a few studies have applied this approach to the analysis of rare eQTLs. Several eQTL studies employed set-based approaches including testing gene expression with multiple single nucleotide polymorphisms (SNPs) chosen by variable selection [16,17] using a gene-based partial least-squares method to correlate multiple gene transcript probes with multiple SNPs [18], and identifying variants associated with transcript and protein modules [19]. These applications were not focused on rare variants, but still afforded higher power with a potential to find significant associations with low-frequency variants.

Few studies have applied a set-based eQTL method for rare variants. Recently, Lutz et al. applied burden and set-based (sequence) kernel association (SKAT) tests to normalize read counts in RNA-sequence (RNA-seq) studies [20]. In this study, we performed a gene-based cis-eQTL analysis using expression data derived from human blood and brain tissue to identify genes that contain a set of potentially regulatory low-frequency and rare variants (minor allele frequency (MAF) < 0.05) that are significantly associated with their expression. Although this design focused on rare variants, and thus has low power to detect expression differences between AD cases and controls, the set-based method can potentially discriminate AD-related targets among a group of genes located within 1 Mb from the expression single nucleotide polymorphisms (eSNPs) that were previously associated with the risk of AD. We also applied a pathway-based approach to determine which genes contribute most to the overall gene expression profile of a significant pathway containing a set of co-expressed functionally related genes.

## 2. Materials and Methods

### 2.1. Study Cohorts

The Alzheimer’s Disease Neuroimaging Initiative (ADNI) is a multisite longitudinal study that began enrolling subjects in 2004, and includes persons with AD, mild cognitive impairment (MCI), and normal cognitive functioning [21]. Affymetrix Human Genome U219 array gene expression data derived from whole blood, whole-genome sequence (WGS) data, and phenotype data were downloaded from a public-access database (http://www.loni.usc.edu (accessed on 11 December 2018)). The portion of the sample included in this study included 207 AD cases, 284 MCI cases, 194 controls, and 28 individuals with missing dementia status.

The Religious Orders Study (ROS)/Memory and Aging Project (MAP) also contributed to this research. ROS enrolled older nuns and priests from across the US without known dementia for a longitudinal clinical analysis and brain donation. MAP enrolled older subjects without dementia from retirement homes, who agreed to brain donation at the time of death [22,23]. RNA-sequence data, including gene expression information derived from dorsolateral prefrontal cortex area tissue donated by 475 participants (281 autopsy-confirmed AD cases and 194 controls), as well as WGS data included in this study, were obtained from the AMP-AD knowledge portal (https://www.synapse.org/#!Synapse:syn3219045 (accessed on 1 July 2018)) [24]. Characteristics of subjects from both cohorts are provided in Table 1.

### 2.2. Data Processing

ADNI microarray gene expression data were normalized and log-transformed using limma [25]. ROSMAP RNA-seq data were normalized and then log-transformed using a previously described pipeline [26]. The log-transformed expression data were evaluated using surrogate variable analysis (SVA) [27] to obtain surrogate variables for global technical effects and hidden effects, which were included as covariates in the analysis models for eQTL discovery. Additional filtering steps of GWAS and gene expression data included eliminating 167 ROSMAP and 96 ADNI subjects with missing data (resulting in the sample sizes reported in Table 1), restricting gene expression data to protein-coding genes (12,971 genes in ROSMAP and 16,025 genes in ADNI), and selecting only bi-allelic low-frequent and rare variants (MAF ≤ 0.05) with a variant call rate of >95%.

### 2.3. Functional Annotation of Variants

Variants in the ADNI and ROSMAP WGS datasets were annotated using CADD v1.6 [28] and GWAVA v1.0 software [29]. Combined Annotation-Dependent Depletion (CADD) scores prioritize functional, deleterious, and disease-causal coding and non-coding variants by integrating multiple annotations into one score by contrasting variants that survived natural selection with simulated mutations [28]. A scaled CADD score of 10 or greater indicates a raw score in the top 10% of all possible reference genome single nucleotide variants (SNVs), and a score of 20 or greater indicates a raw score in the top 1% [28]. Genome-Wide Annotation of Variants (GWAVA) scores predict the functional impact of non-coding genetic variants based on annotations of non-coding elements and genome-wide properties, such as evolutionary conservation and GC-content, in the range of 0–1 with mutations scored >0.5 identified as “functional” and those scored ≤0.5 as “non-functional” [29]. Genomic coordinates of variants in the ADNI dataset that were established using genome build GRCh38 were converted to build hg19 using liftOver software (https://genome.ucsc.edu/cgi-bin/hgLiftOver (accessed on 3 November 2018)). Both ADNI and ROSMAP WGS variants were matched by chromosome, position, reference, and alternate alleles. Variants having a CADD score >15 or a GWAVA region score >0.5 were annotated as having a potential regulatory function.

### 2.4. Set-Based eQTL Analysis

**Figure 1:**
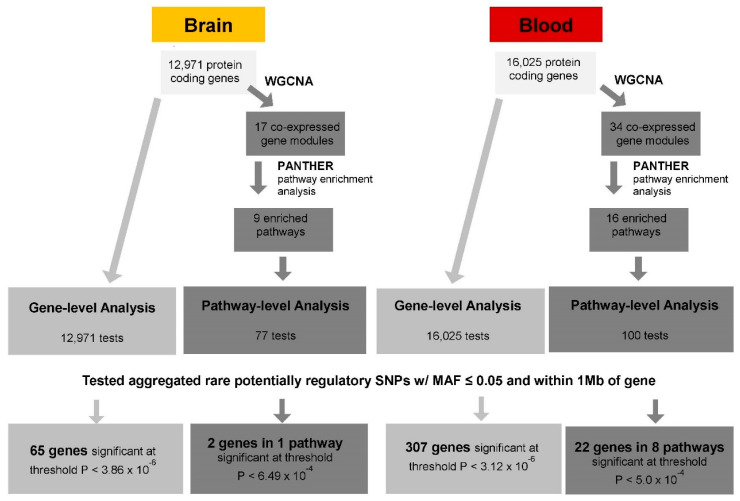
Overview of set-based rare expression quantitative trait loci (eQTL) analysis. Gene-level tests were performed for each protein-coding gene using an aggregate of all potentially regulatory single nucleotide polymorphisms (SNPs) with minor allele frequency ≤0.05 within 1 Mb of each gene. Pathway-level analysis was carried out in two steps. First, the weighted gene co-expression network analysis (WCGNA) method was applied to identify co-expressed gene modules. Next, pathway enrichment analysis was conducted using the Protein Analysis Through Evolutionary Relationships (PANTHER) tool to identify significantly enriched pathways in these gene modules, and pathway-level tests were then performed on each enriched pathway, including the aggregated SNPs for each gene in the module. Results were considered significant (*p* < 0.05) after applying a Bonferroni correction.

The sequence of steps to identify set-based eQTLs in the blood and brain is shown in Figure 1.

#### 2.4.1. Gene-Level cis-eQTL Analysis

For common variants, eQTL analysis entails testing the association of expression of one gene with one variant. Gene-level eQTL analysis was performed by testing the association of expression of one gene with aggregated cis-regulatory variants, limited to those with a frequency of <0.05 and located in or within 1 Mb of the gene. Gene-based tests were performed using the SKAT-O method, which combines the variance component (SKAT) approach and burden tests into one test with optimal power [30]. We implemented SKAT-O tests for set-based eQTL analysis by considering the gene expression value as the outcome, with the aggregated rare variant count as the predictor. The regression model for analyses of the ROSMAP data also included covariates for age, sex, post-mortem interval (PMI), study (ROS or MAP), and a term for a surrogate variable (SV1), derived from the gene expression data matrix to account for unmeasured/hidden technical effects on gene expression using surrogate variable analysis (SVA) [27]. Model covariates for analyses of the ADNI data included baseline age, sex, RNA integrity number (RIN), year of blood sample collection, and SV1. SKAT-O was implemented with group-wise tests using EPACTS software (https://genome.sph.umich.edu/wiki/EPACTS (accessed on 9 March 2021)) with the following parameter specifications: epacts group—vcf [specific chr genome vcf.gz file]\—groupf [file of aggregated rare variants]—out [out file]\—ped [gene expression file]—max-maf 0.05\—pheno $gene—cov Age_baseline—cov Sex—cov RIN—cov Year of Collection—cov SV1—test skat—skat-o—run 8. The significance threshold after adjusting for the number of genes tested was 3.86 × 10^−6^ (0.05/12,971) for analyses of the ROSMAP data and 3.12 × 10^−6^ (0.05/16,024) for analyses of the ADNI data (Figure 1). To identify sentinel variants that contribute the majority of the evidence for significant gene-based results, eQTL tests were performed for all significant genes and each individual potentially regulatory rare variant (MAF ≤ 0.05) within 1Mb of the gene using linear regression models with the above covariates in R [31] for each cis-regulatory variant. The significance threshold after adjusting for the number of unique gene-SNP eQTLs was 1.83 × 10^−6^ (0.05/27,393) for analyses of the ROSMAP data and 1.17 × 10^−7^ (0.05/425,995) for analyses of the ADNI data.

#### 2.4.2. Pathway-Level cis-eQTL Analysis

Pathway-level eQTL analysis was employed to test the association of a pathway, containing many genes, with sets of variants in each of the genes in the pathway one at a time. First, modules of co-expressed genes were identified using the Weighted Gene Co-expression Network Analysis (WGCNA) method implemented in R [32], including all protein-coding genes that were expressed in the ADNI and ROSMAP datasets. Analyses were conducted using the default parameters (soft-threshold power β = 6.00, deepSplit = 2 (medium sensitivity), a minimum module size of 20, and a merge cut height of 0.15) that were recommended by the developers of the software [32] and applied in another AD study [33]. Each gene module can be summarized quantitatively by a module eigengene (ME) value derived from principal component analysis. The ME is considered to be representative of gene expression profiles in a gene module. Next, gene-set pathway enrichment analysis was performed using the Protein Analysis Through Evolutionary Relationships (PANTHER) software tool [34] to determine which pathways were significantly enriched in the gene modules identified from the WGCNA for pathway-level eQTL analysis. Significance of the enriched pathways was determined by the Fisher’s Exact test with a false discovery rate (FDR) of <0.05. Pathway-level eQTL analysis was performed for each significantly enriched pathway. The association of the ME value and each gene in the module was tested individually using all potentially regulatory rare cis-SNPs (MAF < 0.05). Models included the same covariates and parameter specifications as described for the gene-level eQTL tests and were analyzed using the SKAT-O method implemented in EPACTS. A total of 77 genes in 9 enriched pathways were evaluated in the ROSMAP dataset, and 100 genes in 16 enriched pathways were evaluated in the ADNI dataset. After correction for the number of genes that were tested, the thresholds for significant pathway-level rare eQTLs were *p* < 6.49 × 10^−4^ in the ROSMAP dataset and *p* < 5.0 × 10^−4^ in the ADNI dataset (Figure 1).

#### 2.4.3. Comparison of Rare and Common eQTLs

To determine whether both common variants and gene-level aggregated rare/low-frequency variants target expression of the same genes, we evaluated the overlap in significant gene-based cis-eQTLs with those involving common variants (MAF > 0.05) within 1 Mb of protein-coding genes that were obtained previously from the Framingham Heart Study (blood) and ROSMAP (brain) gene expression datasets [26]. These comparisons not only indicated which eGenes are regulated by rare and/or common variants, but also determined whether multiple variants can separately up- or down-regulate expression of the same gene.

## 3. Results

### 3.1. Gene-Level eQTL Associations

In the gene-level eQTL analysis, aggregating on average 416 unique low-frequency and rare variants for each gene, 65 significant gene-level eQTLs (*p* < 3.86 × 10^−6^) were identified in the brain (Figure 1, Table S1). Eight of these genes, including established AD genes *HLA-DRB1* [35] and *HLA-DRB5* [36], are located in or near the major histocompatibility locus. By comparison, 307 significant gene-level eQTLs, with an average of 678 unique variants, were observed in blood at *p* < 3.12 × 10^−6^ (Figure 1, Table S2). Among these genes, *ABCA7*, *ECHDC3*, and *MS4A6A* are known AD loci [35,36]. The genes *GNMT*, *LDHC*, *RBPMS2*, *DUS2*, and *HP* were significant in both the brain and blood (Table 2), noting that the evidence for *RBPMS2* was stronger in the blood (*p* = 1.69 × 10^−36^) than the brain (*p* = 9.90 × 10^−8^).

### 3.2. Variant-Level eQTL Associations

Examination of the variant-level eQTL associations for the 65 significant genes in the brain identified 61 significant eGene-eSNP eQTL pairs, involving 22 unique eGenes (Table S3). By a very wide margin, the most significant eQTL pair featured rs772849040 located in *NFAT5*, which targeted *DDX19A-DDX19B* (*p* ≤ 1.0 × 10^−314^). *DDX19A-DDX19B* was also a significant eGene for rs17881635 located in COG4 (*p* = 6.26 × 10^−23^). *COPZ1* and *TMPRSS6* were both significant eGenes for seven eSNPs each. A much larger number of eQTL pairs (*n* = 832) were significant in the blood, in which 185 eGenes were unique (Table S4). Four of these genes had 20 or more significant eSNPs: *KRT79* (*n* = 36), *TAC3* (*n* = 32), *CDK12* (*n* = 24), and *SOS1* (*n* = 20). *LDHC* was a significant eGene for two eSNPs in the blood (rs117652970, *p* = 1.12 × 10^−21^ and rs17579565, *p* = 8.26 × 10^−21^) and a third eSNP in the brain rs773835421, *p* = 1.60 × 10^−6^). Adjacent genes *DHRS4* and its homolog *DHRS4L2* were significant eGene targets for 17 eSNPs. Similarly, three SNPs were each significant eQTLs paired with *ATP6V0D1* and *CMTM2*, and four SNPs were each significant eQTLs paired with *IKZF3* and *GSDMA*. In the brain, rs1260874991 and rs1405001784 were significant eSNPs for two zinc finger protein genes (*ZNF101* and *ZNF103*).

### 3.3. Pathways Enriched in the Brain and Blood

Pathway enrichment analysis of each gene module revealed 9 significant enriched pathways in the brain and 16 in the blood (Table 3). The apoptosis signaling, cholecystokinin receptor (CCKR) signaling map, and inflammation mediated by chemokine and cytokine signaling pathways were enriched in both the brain and blood. Focusing on genes in the significantly enriched pathways in the brain, the aggregated rare variants in *CCL7* and *CCL8* were associated with the inflammation mediated by chemokine and cytokine signaling pathway (*p* = 1.84 × 10^−5^ and *p* = 4.50 × 10^−4^, respectively, Table 4). In total, 6 of the 22 genes that contained significant aggregated rare eQTLs associated with pathway expression in the blood were members of the same inflammation pathway: *ALOX5AP* (*p* = 1.26 × 10^−4^), *CXCR2* (*p* = 1.53 × 10^−6^), *FPR2* (*p* = 1.25 × 10^−4^), *GRB2* (*p* = 6.04 × 10^−7^), *IFNAR1* (*p* = 1.98 × 10^−5^), and *RAF1* (*p* = 2.11 × 10^−5^) (Table 4). Furthermore, *CFLAR* (*p* = 2.42 × 10^−4^), *TMBIM6* (*p* = 4.48 × 10^−4^), and *TNFRSF10C* (*p* = 8.77 × 10^−5^) were significant rare variant eQTLs in apoptosis signaling pathways in the blood. Significant aggregated rare variant eQTLs were observed with *ALOX5AP* in both gene-level (*p* = 2.20 × 10^−10^) and pathway-level (*p* = 1.26 × 10^−4^) analyses (Table 4).

### 3.4. Gene Targets of eQTLs in the Brain and Blood

**Figure 2:**
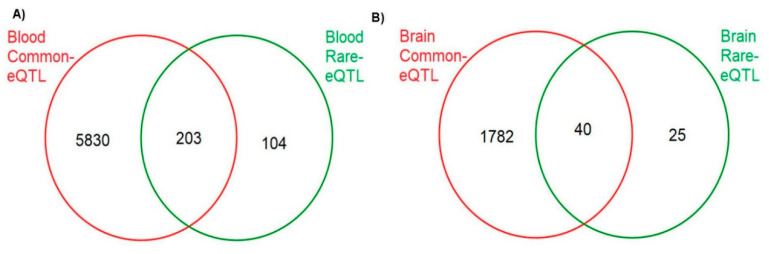
Overlap of significant genes in rare gene-level and common eQTLs in (**A**) the blood and (**B**) the brain.

Comparison of significant rare and common eQTLs in each tissue (Figure 2) revealed 203 genes in the blood and 40 genes in the brain that were targets of rare and common eSNPs (Table S5), including 19 in the blood and 9 in the brain that have both been previously implicated in AD (Table 5). Three genes (*LDHC*, *RBPMS2*, and *HP*) are targets that were observed in significant rare and common eQTLs in the brain and the blood.

## 4. Discussion

Our study demonstrates that low-frequency and rare variants have a significant impact on both the expression of genes considered individually and the co-expression of genes in pathways. Our study highlights the value of the set-based rare-eQTL method because, similar to gene-based association tests, many novel significant genes we identified were not detected by the analysis of rare variants individually, which requires a much larger sample size. In addition, many of the most significant rare-variant findings involved genes with prior connections to AD through case-control comparisons using GWAS, gene expression, and functional studies.

Several of the most significant gene-level eQTL findings in the blood have previously been implicated in AD. *MS4A6A* (*p* = 1.77 × 10^−22^) is among a family of genes containing many SNPs that are associated with AD risk at the genome-wide level [35,36]). A meta-analysis of gene expression studies found that *NUMA1* (*p =* 6.01 × 10^−76^) was significantly upregulated in the hippocampus of AD cases [60], and another study showed that downregulation of *GAD1* (*p =* 1.49 × 10^−58^) was associated with reduced neuronal activity [61]. Follistatin, encoded by *FST* (*p* = 4.02 × 10^−30^), is a gonadal protein that inhibits the follicle-stimulating protein. The transmembrane protein, tomoregulin-2, contains follistatin-like modules and is found extensively in amyloid plaques in AD brains [62]. *KIF1B* (*p =* 4.49 × 10^−21^) expression is significantly increased in AD and is associated with accelerated progression in neurodegenerative diseases [50,51]. The established AD gene *ADAM10* [35] is downregulated by *SFRP1* (*p =* 2.16 × 10^−20^), which is significantly increased in the brain and cerebrospinal fluid (CSF) of AD patients [63]. *EXOC2* (*p =* 6.19 × 10^−9^) was identified as an AD age-of-onset modifier [64] and contains a rare missense variant that was observed in seven AD cases in an AD whole-exome sequencing study [9].

Four of the five significant gene-level rare eQTLs in the brain and blood (Table 1) have also been implicated in AD. *GNMT* expression has been detected in the hippocampus and its deficiency results in reduced neurogenic capacity, spatial learning, and memory impairment [65]. *LDHC* has differentially methylated regions in the blood in AD cases [66]. The overexpression of *DUS2* reduces Aβ_42_ toxicity [67]. The acute-phase protein haptoglobin, encoded by *HP*, is significantly elevated among AD patients compared to healthy controls in serum [44,68] and CSF [69] in Asians and persons of European ancestry. The *HP* 1/1 genotype was associated with poorer cognitive function and greater cognitive decline than other *HP* genotypes in a sample of 466 African Americans with type 2 diabetes [70]. The RNA-binding protein *RBPMS2* has not been linked to AD but is a constituent of a leukocyte signature for traumatic brain injury [71].

We identified several pathways that are significantly enriched with genes involved in the CCKR signaling map, apoptosis signaling, and inflammation mediated by chemokine and cytokine signaling pathways, all of which have been linked to AD [72,73,74]. Wnt signaling, one of the significant pathways we observed in brain, suppresses tau phosphorylation and Aβ production/aggregation, inhibits *BACE1* expression, and promotes neuronal survival [75]. *HSPA5* (*p* = 7.91 × 10^−5^), one of the significant pathway-level eQTL findings, is involved in both amyloid precursor protein metabolism and neuronal death in AD [76].

Our rare-eQTL gene-level and pathway-level results confirm the substantial immune and inflammatory component to AD. Significant gene-level rare eQTLs in the brain included several HLA region loci linked to AD by GWAS (*HLA-DRB1* and *HLA-DRB5* [35,36]) and cell-type specific eQTL analysis (*HLA-DOB* [26]). *IL27* (*p* = 1.69 × 10^−30^) is a cytokine, and *CARD17* (*p =* 6.73 × 10^−13^) encodes a regulatory protein of inflammasomes, which are responsible for the activation of inflammatory responses [77]. Overall, 8 of the 21 significant pathway-level rare eQTLs involved genes which have roles in the inflammation mediated by the chemokine and cytokine signaling pathway. Chemokine levels were found to be significantly increased in serum, CSF, and brain tissue from AD cases [78]. Chemokine receptor *CXCR2* induces Aβ peptides [79]. Another gene in this group, *IFNAR1*, encodes the interferon α and β receptor subunit 1. Primary microglia isolated from the brains of *APP/PS1* mutant mice with ablated type-I interferon signaling have shown reduced levels of Aβ_1–42_ [80]. In addition to being a significant pathway-level rare eQTL, *FPR2* is also very significant eQTL in the blood (*p =* 1.22 × 10^−240^), and more specifically, in interferon and anti-bacterial cells (*p =* 3.81 × 10^−17^) [26]. It is involved in the uptake and clearance of Aβ and contributes to innate immunity and inflammation [81]. *ALOX5AP* (a.k.a. *FLAP)* is expressed in microglia and encodes a protein which, with 5-lipoxygenase, is required for leukotriene synthesis. Leukotrienes are arachidonic acid metabolites which have been implicated in neuroinflammatory and amyloidogenesis processes in AD [82]. Pharmacological inhibition of FLAP in Tg2576 mice significantly reduced tau phosphorylation at multiple sites and increased post-synaptic density protein-95 and microtubule-associated protein 2 [83]. Growth factor receptor-bound protein 2, encoded by *GRB2,* is an adaptor protein that is involved in the trafficking of Aβ [84]. Although the inflammation pathway was implicated in the eQTL analysis in both the brain and blood, our results showed that the genes significantly contributing to pathway expression differed between the tissues. This suggests that AD-related inflammatory processes may differ in the blood and brain.

We observed significant eQTLs involving 27 target genes, previously implicated in AD through genetic and experimental approaches, which were paired with rare variants identified in this study and previously reported common variants [26] (Table 5). *HP* was the only gene in this group whose expression was influenced by rare and common eSNPs in both the blood and brain, and thus, it has notable potential as a blood-based biomarker reflecting AD-related gene expression changes in brain.

Although the set-based rare-eQTL method employed in this study has multiple strengths in comparison to the analysis of individual rare eQTLs (e.g., higher power, reduced multiple testing burden, and ability to detect the effects of variants with lower frequency), our results should be interpreted cautiously in light of several limitations. Comparisons between the brain and blood were not conducted using data from the same subjects, and thus may underestimate similarities across tissues. Also, brain expression patterns may reflect post-mortem changes unrelated to disease or cell-type specific expression [85]. The set-based method using SKAT-O allows for opposite effect directions of the constituent SNPs in the test; however, closer scrutiny of the individual SNPs is necessary to draw conclusions about the collective influence of rare variants on expression, as well as consistency of the effect direction across tissues. Our results, which were generated from analyses at the tissue level, do not account for patterns that are cell-type specific within the blood and brain, as we recently demonstrated for common individual variant eQTLs in these datasets [26]. In addition, it is unclear whether the set-based eQTL method applied in this study would behave similarly for rare (MAF < 0.01) and low-frequency (0.01 < MAF < 0.05) variants analyzed separately. Finally, although this investigation was conducted using tissue obtained from participants enrolled in studies of AD, the direct testing of the relevance of findings from the set-based tests of rare variants to AD status was not feasible, because the sample size was insufficient to have representation of the sentinel variants in both the case and control groups. This limitation is analogous to the difficulty encountered in the replication of the aggregated rare variant test findings in AD genetic association studies [7,8]. Thus, further studies of some genes are needed to establish their role in AD. Nonetheless, our study provided evidence favoring specific genes under previously established AD-association peaks whose expression may be differentially or concordantly regulated in the blood and brain (Table 5).

## 5. Conclusions

This study of gene-based and pathway-level rare eQTLs implicated novel genes that may have important roles in AD, found additional evidence supporting the contribution of immune/inflammatory pathways in AD, and demonstrated the utility of a set-based eQTL approach for assessing the role of rare variants in molecular mechanisms underlying the disease. The relevance of these findings to AD should be validated in larger samples with sufficient power for comparing patterns between AD cases and controls, as well as with functional experiments.

## Figures and Tables

**Table 1:** Characteristics of subjects in the Religious Orders Study/Memory and Aging Project (ROSMAP) and Alzheimer’s Disease Neuroimaging Initiative (ADNI) datasets.

Dataset	Race	N	AD Cases	MCI Cases	Controls	Female	Age *
ROSMAP (Brain)	NHW 98% AA 2% Other <0.01%	475	281	0	194	63%	85.9 (4.8)
ADNI (Blood)	NHW 93% AA 4% Other 3%	713	207	284	222	44%	76.3 (8.1)

NHW—non-Hispanic white, AA—African American. * mean (standard deviation).

**Table 2:** Significant gene-level eQTLs common to blood and brain.

Chr	Begin Position	End Position	Gene	Brain			Blood		
				CVar +	Unique Var ^	*p*-Value	CVar +	Unique Var ^	*p*-Value
6	41,942,338	43,929,364	*GNMT*	671	437	1.85 × 10^−6^	1006	640	2.87 × 10^−7^
11	17,434,230	19,468,040	*LDHC*	429	273	2.07 × 10^−7^	762	473	2.25 × 10^−10^
15	64,039,999	66,063,761	*RBPMS2*	404	249	9.90 × 10^−8^	648	417	1.69 × 10^−36^
16	67,034,867	69,106,452	*DUS2*	714	482	1.98 × 10^−6^	1085	723	6.41 × 10^−08^
16	71,090,452	73,094,829	*HP*	741	461	2.28 × 10^−9^	1206	750	2.43 × 10^−11^

+ Cumulative number of variants. ^ Number of unique variants. Chromosome and map position according to GRCh37 assembly.

**Table 3:** Significant pathway enrichment in gene modules in the brain and blood.

Pathway	# Genes in Pathway	Gene Module	# Module Genes in Pathway	Module Genes			Uncorrected *p*-Value	FDR
				Expected # of Genes *	Fold Enrichment ^†^	+/−		
BRAIN								
Apoptosis signaling	77	7	12	1.64	7.3	+	3.09 × 10^−7^	5.01 × 10^−5^
Toll receptor signaling	32	8	6	0.46	12.97	+	1.45 × 10^−5^	2.36 × 10^−3^
Wnt signaling	235	4	21	7.35	2.86	+	3.49 × 10^−5^	5.65 × 10^−3^
Cadherin signaling	127	4	14	3.97	3.53	+	9.59 × 10^−5^	7.77 × 10^−3^
CCKR signaling map	111	7	10	2.37	4.22	+	2.22 × 10^−4^	1.20 × 10^−2^
Gonadotropin-releasing hormone receptor	152	4	14	4.75	2.95	+	5.28 × 10^−4^	2.14 × 10^−2^
p53	62	7	7	1.32	5.29	+	5.76 × 10^−4^	2.33 × 10^−2^
Inflammation mediated by chemokine and cytokine signaling	173	16	5	0.51	9.89	+	1.54 × 10^−4^	2.50 × 10^−2^
Angiogenesis	126	4	12	3.94	3.05	+	9.95 × 10^−4^	3.22 × 10^−2^
BLOOD								
Blood coagulation	43	24	8	0.27	29.9	+	8.22 × 10^−10^	1.34 × 10^−7^
Parkinson’s disease	85	15	7	0.79	8.82	+	2.28 × 10^−5^	3.72 × 10^−3^
Inflammation mediated by chemokine and cytokine signaling	237	14	11	2.27	4.84	+	2.50 × 10^−5^	4.08 × 10^−3^
T-cell activation	73	32	4	0.18	22.02	+	3.87 × 10^−5^	6.30 × 10^−3^
B-cell activation	66	12	6	0.66	9.08	+	7.89 × 10^−5^	1.29 × 10^−2^
PDGF signaling	127	12	7	1.27	5.5	+	3.79 × 10^−4^	2.06 × 10^−2^
Apoptosis signaling	112	5	12	3.15	3.81	+	1.51 × 10^−4^	2.47 × 10^−2^
JAK/STAT signaling	17	7	4	0.28	14.33	+	3.21 × 10^−4^	2.62 × 10^−2^
Ras	64	5	8	1.8	4.44	+	7.61 × 10^−4^	3.10 × 10^−2^
CCKR signaling map	164	5	14	4.61	3.04	+	3.94 × 10^−4^	3.21 × 10^−2^
Angiotensin II-stimulated signaling through G proteins and β-arrestin	33	5	6	0.93	6.47	+	6.14 × 10^−4^	3.34 × 10^−2^
Histamine H2 receptor-mediated signaling	24	5	5	0.67	7.41	+	1.03 × 10^−3^	3.37 × 10^−2^
Inflammation mediated by chemokine and cytokine signaling	237	24	7	1.47	4.75	+	7.94 × 10^−4^	4.32 × 10^−2^
Heme biosynthesis	11	6	4	0.25	15.93	+	2.74 × 10^−4^	4.47 × 10^−2^
Integrin signalling	180	7	11	2.96	3.72	+	2.84 × 10^−4^	4.62 × 10^−2^
Inflammation mediated by chemokine and cytokine signaling	237	20	8	1.78	4.48	+	5.07 × 10^−4^	8.26 × 10^−2^

* Number of genes expected in the gene module by chance based on the total set of genes in the pathway as determined for the ROSMAP and ADNI datasets. ^†^ fold enrichment = # module genes in pathway/expected number of genes in module.

**Table 4:** Significant pathway-level eQTLs in the brain or blood by aggregating cis rare variants.

CHR	Begin Position	End Position	Gene	CVAR +	Unique VAR ^	*p*-Value	Gene Module	Pathway
17	31,600,172	33,592,552	*CCL7* *	340	206	1.84 × 10^−5^	16	Inflammation mediated by chemokine and cytokine signaling
17	31,648,819	33,621,655	*CCL8* *	319	195	4.50 × 10^−4^	16	Inflammation mediated by chemokine and cytokine signaling
17	72,322,351	74,401,630	*GRB2*	1108	717	6.04 × 10^−7^	14	Inflammation mediated by chemokine and cytokine signaling
2	217,992,496	220,001,949	*CXCR2*	943	564	1.53 × 10^−6^	14	Inflammation mediated by chemokine and cytokine signaling
5	174,085,268	176,108,976	*HRH2*	335	196	9.07 × 10^−6^	5	Histamine H2 receptor mediated signaling
1	25,859,096	27,901,441	*RPS6KA1*	1208	790	1.01 × 10^−5^	5	Ras Pathway, CCKR signaling map
11	76,033,278	78,180,311	*PAK1*	565	355	1.83 × 10^−5^	5	Ras Pathway, CCKR Signaling map
21	33,696,834	35,718,581	*IFNAR1*	525	332	1.98 × 10^−5^	14	Inflammation mediated by chemokine and cytokine signaling
3	11,628,812	13,702,170	*RAF1*	431	255	2.11 × 10^−5^	14	Inflammation mediated by chemokine and cytokine signaling
1	83,964,144	85,961,982	*GNG5*	589	336	3.43 × 10^−5^	5	Histamine H2 receptor mediated signaling
9	115,150,150	117,160,754	*ALAD*	620	425	4.97 × 10^−5^	6	Heme biosynthesis
1	44,478,672	46,476,606	*UROD*	1061	649	5.92 × 10^−5^	6	Heme biosynthesis
19	13,202,507	15,228,794	*PRKACA*	767	501	7.60 × 10^−5^	5	Histamine H2 receptor mediated signaling, CCKR signaling map
9	127,005,465	128998618	*HSPA5*	1235	692	7.91 × 10^−5^	15	Parkinson disease
8	21,946,761	23968794	*TNFRSF10C*	817	520	8.77 × 10^−5^	5	Apoptosis signaling
19	51,273,985	53272173	*FPR2*	440	280	1.23 × 10^−4^	14	Inflammation mediated by chemokine and cytokine signaling
13	30,317,837	32,332,540	*ALOX5AP*	297	197	1.26 × 10^−4^	14	Inflammation mediated by chemokine and cytokine signaling
2	200,984,212	203,030,077	*CFLAR*	637	404	2.42 × 10^−4^	5	Apoptosis signaling
17	39,458,200	41,463,831	*STAT5A*	1093	708	3.08 × 10^−4^	12	PDGF signaling
14	50,190,597	52,294,891	*NIN*	516	323	3.65 × 10^−4^	12	PDGF signaling
12	49,108,257	51,158,233	*TMBIM6*	1082	716	4.48 × 10^−4^	5	Apoptosis signaling

+ Cumulative number of variants; ^ Number of unique variants; * Results from brain (otherwise results from blood); Chromosome and map position according to GRCh37 assembly.

**Table 5:** Genes previously implicated in AD whose expression is influenced by both rare and common SNPs.

eQTLs in Blood		eQTLs in Brain	
eGene	Reference	eGene	Reference
*ABCA7* *	[36]	*ACOT1*	[37]
*ADAMTSL4*	[38]	*HLA-A*	[39]
*ARRB2*	[40]	*HLA-DOB* *	[26]
*ATG7*	[41]	*HLA-DRB1* *	[35,41]
*CD36*	[42]	*HLA-DRB5* *	[36]
*CREB5*	[43]	*HP*	[44]
*CTNNAL1*	[45]	*POMC*	[46]
*ECHDC3* *	[35]	*RNF39*	[47,48]
*HP*	[44]	*ZNF253*	[49]
*KF1B*	[50,51]		
*LRRC2*	[52]		
*MS4A6A* *	[36]		
*PADI2*	[53]		
*PDLIM5*	[54]		
*S100A12*	[55]		
*SPPL3*	[56]		
*TMEM51*	[57]		
*TREML4*	[58]		
*UBE4B*	[59]		

* AD locus established by GWAS.

## Data Availability

The results published here are in whole or in part based on data obtained from the AD Knowledge Portal (https://adknowledgeportal.synapse.org (accessed on 1 July 2018)). ROSMAP study data were provided by the Rush Alzheimer’s Disease Center, Rush University Medical Center, Chicago. ADNI data can be obtained by study investigators (adni.loni.usc.edu (accessed on 9 March 2021)).

## Supplementary Materials

The following are available online at https://www.mdpi.com/2073-4425/12/3/419/s1, Table S1: Gene-level rare cis-eQTLs in the brain (*p* < 3.86 × 10^−6^), Table S2: Individual SNP eQTLs in the brain (*p* < 1.83 × 10^−6^), Table S3: Gene-level rare cis-eQTLs in the blood (*p* < 3.12 × 10^−6^), Table S4: Individual SNP eQTLs in the blood (*p* < 1.17 × 10^−7^), Table S5: eGene targets of both rare and common eQTLs in the blood and brain.

## Abbreviations

Aβ	amyloid-β
AD	Alzheimer disease
ADNI	Alzheimer’s Disease Neuroimaging Initiative
eQTL	Expression quantitative trait locus
CADD	Combined annotation-dependent depletion
GWAVA	Genome-wide annotation of variants
MAF	Minor allele frequency
MCI	Mild cognitive impairment
ME	module eigengene
PANTHER	Protein Analysis Through Evolutionary Relationships
ROSMAP	Religious Orders Study/Memory and Aging Project
SNP	Single nucleotide polymorphism
SVA	Surrogate variable analysis
WCGNA	Weighted gene co-expression network analysis
WGS	Whole-genome sequence
