# Supplementary material for: Set-Based Rare Variant Expression Quantitative Trait Loci in Blood and Brain from Alzheimer Disease Study Participants
Source: Genes (Basel). 2021 Mar 15;12(3):419. doi: 10.3390/genes12030419 (PMC7999141; doi:10.3390/genes12030419)
Supplement: Supplementary file 1 [file genes-12-00419-s001.zip › rare-eqtl paper Supp revised - Genes 3-4-21.docx]

**Supplementary Table 1.** Gene-level rare cis-eQTLs in brain (P < 3.86x10^-6^)

| **CHR** | **Begin Pos** | **End Pos** | **CVAR ^+^** | **Unique VAR^** | **P-value** | **Gene** |
| --- | --- | --- | --- | --- | --- | --- |
| 5 | 22985688 | 25142892 | 177 | 113 | 4.56x10^-49^ | *C5orf17* |
| 16 | 27512608 | 29486403 | 269 | 169 | 1.69x10^-30^ | *IL27* |
| 6 | 29051606 | 31043360 | 426 | 220 | 7.71x10^-27^ | *RNF39* |
| 19 | 18779631 | 20736620 | 248 | 173 | 2.19x10^-22^ | *ZNF101* |
| 1 | 147741039 | 149755790 | 186 | 69 | 2.42x10^-21^ | *NBPF16* |
| 4 | 89823539 | 91848018 | 153 | 96 | 1.49x10^-19^ | *MMRN1* |
| 19 | 18977711 | 20990019 | 221 | 155 | 7.45x10^-19^ | *ZNF253* |
| 1 | 25378602 | 27385983 | 482 | 320 | 1.96x10^-16^ | *TRIM63* |
| 9 | 138266509 | 140255330 | 553 | 352 | 7.16x10^-16^ | *DNLZ* |
| 7 | 43155135 | 45148545 | 252 | 162 | 1.56x10^-13^ | *POLD2* |
| 2 | 169503264 | 171547270 | 343 | 205 | 1.85x10^-13^ | *CCDC173* |
| 11 | 103966694 | 105967614 | 107 | 68 | 6.73x10^-13^ | *CARD17* |
| 14 | 73016786 | 75002697 | 423 | 267 | 2.34x10^-12^ | *ACOT1* |
| 16 | 13847527 | 15857759 | 298 | 191 | 1.64x10^-11^ | *NPIPA2* |
| 1 | 149958668 | 151965171 | 540 | 352 | 1.83x10^-11^ | *ANXA9* |
| 4 | 43689060 | 45457518 | 69 | 40 | 2.49x10^-11^ | *GUF1* |
| 16 | 69333666 | 71398570 | 474 | 316 | 3.54x10^-11^ | *RP11-529K1.3* |
| 15 | 81558119 | 83561556 | 184 | 106 | 6.95x10^-11^ | *FAM154B* |
| 1 | 53492087 | 55481065 | 460 | 281 | 1.06x10-_10_ | *LDLRAD1* |
| 7 | 127418124 | 129414750 | 519 | 310 | 1.07x10_-10_ | *OPN1SW* |
| 6 | 27300777 | 29323838 | 369 | 221 | 1.22x10_-10_ | *ZSCAN31* |
| 14 | 73783107 | 75825914 | 482 | 303 | 1.87x10^-10^ | *VRTN* |
| 19 | 9406430 | 11373256 | 444 | 293 | 1.87x10^-10^ | *ICAM4* |
| 6 | 29051606 | 31002490 | 425 | 219 | 2.38x10^-10^ | *ZNRD1* |
| 2 | 24400738 | 26360191 | 286 | 180 | 2.72x10^-10^ | *POMC* |
| 16 | 17417023 | 19440804 | 267 | 149 | 4.61x10^-10^ | *NPIPA8* |
| 6 | 31783750 | 33784748 | 926 | 480 | 1.24x10^-9^ | *HLA-DOB* |
| 2 | 60375560 | 62373811 | 538 | 337 | 1.65x10^-9^ | *C2orf74* |
| 12 | 53696863 | 55726368 | 853 | 532 | 1.81x10^-9^ | *COPZ1* |
| 16 | 71090452 | 73094829 | 741 | 461 | 2.28x10^-9^ | *HP* |
| 11 | 112263796 | 114271056 | 464 | 266 | 2.34x10^-9^ | *ANKK1* |
| 19 | 1941163 | 3942240 | 400 | 276 | 1.41x10^-8^ | *ZNF77* |
| 6 | 31496752 | 33476802 | 1053 | 565 | 3.58x10^-8^ | *HLA-DRB5* |
| 1 | 247920924 | 249234675 | 158 | 88 | 8.53x10^-8^ | *LYPD8* |
| 15 | 64039999 | 66063761 | 404 | 249 | 9.90x10^-8^ | *RBPMS2* |
| 12 | 9980142 | 11951867 | 155 | 98 | 1.02x10^-7^ | *TAS2R9* |
| 2 | 95333119 | 96830428 | 153 | 88 | 1.10x10^-7^ | *ZNF514* |
| 1 | 227399406 | 229357415 | 255 | 176 | 1.20x10^-7^ | *C1orf145* |
| 6 | 28909389 | 30908847 | 458 | 238 | 2.02x10^-7^ | *HLA-A* |
| 15 | 42622894 | 44630360 | 522 | 332 | 2.04x10^-7^ | *ADAL* |
| 11 | 17434230 | 19468040 | 429 | 273 | 2.07x10^-7^ | *LDHC* |
| 22 | 41523734 | 43525652 | 369 | 245 | 2.49x10^-7^ | *CYP2D6* |
| 16 | 71090452 | 73110685 | 755 | 468 | 2.80x10^-7^ | *HPR* |
| 1 | 149349077 | 151447295 | 508 | 334 | 3.36x10^-7^ | *RPRD2* |
| 12 | 49721015 | 51789242 | 429 | 284 | 4.37x10^-7^ | *FAM186A* |
| 8 | 26682344 | 28667180 | 197 | 127 | 4.71x10^-7^ | ESCO2 |
| 7 | 63347619 | 65355768 | 132 | 51 | 5.07x10^-7^ | *ZNF273* |
| 6 | 34447206 | 36438023 | 417 | 258 | 5.55x10^-7^ | *RPL10A* |
| 12 | 20680554 | 22677495 | 165 | 112 | 6.47x10^-7^ | *C12orf39* |
| 12 | 9062912 | 11038219 | 163 | 106 | 7.21x10^-7^ | *KLRF2* |
| 2 | 157116105 | 159166135 | 545 | 334 | 8.96x10^-7^ | *GALNT5* |
| 2 | 131958523 | 134009777 | 395 | 157 | 1.04x10^-6^ | *ANKRD30BL* |
| 1 | 39240400 | 41236581 | 492 | 310 | 1.69x10^-6^ | *OXCT2* |
| 14 | 44615125 | 46540143 | 233 | 140 | 1.73x10^-6^ | *PRPF39* |
| 6 | 82920251 | 85135804 | 241 | 160 | 1.79x10^-6^ | *M*E*1* |
| 1 | 44966476 | 46963603 | 600 | 383 | 1.85x10^-6^ | *CCDC163P* |
| 6 | 41942338 | 43929364 | 671 | 437 | 1.85x10^-6^ | *GNMT* |
| 10 | 62424318 | 64520825 | 669 | 426 | 1.95x10^-6^ | *C10orf107* |
| 6 | 31548925 | 33554557 | 1027 | 545 | 1.98x10^-6^ | *HLA-DRB1* |
| 16 | 67034867 | 69106452 | 714 | 482 | 1.98x10^-6^ | *DUS2* |
| 21 | 46707826 | 48118123 | 184 | 132 | 2.06x10^-6^ | *YB*E*Y* |
| 6 | 30104753 | 32098106 | 830 | 448 | 2.49x10^-6^ | *PSORS1C1* |
| 14 | 74522315 | 76532783 | 500 | 308 | 2.53x10^-6^ | *ACYP1* |
| 22 | 36513231 | 38505189 | 446 | 289 | 2.64x10^-6^ | *TMPRSS6* |
| 7 | 35121219 | 37109559 | 238 | 133 | 3.24x10^-6^ | *PP13004* |

**+** Cumulative number of variants

**^** Number of unique variants

Chromosome position according to GRCh37 assembly

**Supplementary Table 3.** Individual SNP eQTLs in brain (P < 1.83 x 10^-6^)

| **Map Position** | **Gene** | **Beta** | **Std Err** | **P-value** | **SNP ID** | **MAF** |
| --- | --- | --- | --- | --- | --- | --- |
| 2:60683420 | *C2orf74* | -2.11 | 0.41 | 2.32x10^-7^ | rs546615129 | 0.0002 |
| 2:60753967 | *C2orf74* | -2.11 | 0.41 | 2.32x10^-7^ | rs568141042 | 0.0004 |
| 5:23132477 | *C5orf17* | 1.16 | 0.2 | 3.25x10^-9^ | rs926892186 | NA |
| 5:24322422 | *C5orf17* | 0.54 | 0.1 | 3.23x10^-8^ | rs527917142 | 0.0006 |
| 11:104480278 | *CARD17* | 0.07 | 0.01 | 1.19x10^-12^ | rs1402267439 | NA |
| 12:53853136 | *COPZ1* | -2.35 | 0.15 | 1.30x10^-54^ | rs764782139 | 0.00001 |
| 12:54367799 | *COPZ1* | -2.35 | 0.15 | 1.30x10^-54^ | rs899379914 | NA |
| 12:54511341 | *COPZ1* | -2.35 | 0.15 | 1.30x10^-54^ | rs978989525 | NA |
| 12:54520272 | *COPZ1* | -2.35 | 0.15 | 1.30x10^-54^ | rs377096982 | 0.00006 |
| 12:54623909 | *COPZ1* | -0.79 | 0.1 | 3.80x10^-15^ | rs140796071 | 0.003 |
| 12:54673746 | *COPZ1* | -0.32 | 0.07 | 1.21x10^-6^ | rs374154317 | 0.0022 |
| 12:54986339 | *COPZ1* | -2.35 | 0.15 | 1.30x10^-54^ | rs1002210370 | NA |
| 16:69600184 | *DDX19A-DDX19B* | 0.39 | 0.01 | <1.0x10^-314^ | rs772849040 | 0.00001 |
| 16:70512234 | *DDX19A-DDX19B* | 0.08 | 0.01 | 6.26x10^-23^ | rs17881635 | 0.0051 |
| 9:139694595 | *DNLZ* | 2.57 | 0.43 | 1.92x10^-9^ | rs767575212 | 0.00003 |
| 9:140082242 | *DNLZ* | 2.18 | 0.44 | 6.08x10^-7^ | rs200629106 | 0.00016 |
| 12:51566637 | *FAM186A* | 0.01 | 0 | 1.59x10^-9^ | rs75532397 | 0.0088 |
| 2:157192762 | *GALNT5* | 0.12 | 0.01 | 1.85x10^-32^ | rs538009405 | 0.0002 |
| 2:157716598 | *GALNT5* | 0.03 | 0.01 | 9.43x10^-8^ | rs180703346 | 0.0006 |
| 2:157861592 | *GALNT5* | 0.03 | 0.01 | 5.11x10^-9^ | rs35961069 | 0.0032 |
| 19:10742166 | *ICAM4* | 1.17 | 0.1 | 6.67x10^-30^ | rs147820753 | 0.0043 |
| 19:10781827 | *ICAM4* | 1.32 | 0.19 | 4.81x10^-12^ | rs369745966 | 0.00004 |
| 19:10952607 | *ICAM4* | 1.28 | 0.19 | 2.62x10^-11^ | NA | NA |
| 19:11034582 | *ICAM4* | 0.24 | 0.05 | 1.30x10^-6^ | rs77275750 | 0.012 |
| 19:11319636 | *ICAM4* | 0.45 | 0.07 | 7.40x10^-11^ | rs117328686 | 0.035 |
| 12:9268393 | *KLRF2* | 0.04 | 0.01 | 1.12x10^-12^ | rs201671036 | 0.0016 |
| 11:17867845 | *LDHC* | 1.07 | 0.22 | 1.60x10_-6_ | rs773835421 | NA |
| 1:54200597 | *LDLRAD1* | 0.03 | 0.01 | 6.58x10_-9_ | rs148563942 | 0.0018 |
| 1:55481065 | *LDLRAD1* | 0.14 | 0.01 | 1.02x10^-42^ | rs1242270816 | NA |
| 7:127560949 | *OPN1SW* | 0.62 | 0.11 | 2.14x10^-8^ | rs568900505 | 0.0002 |
| 7:127668180 | *OPN1SW* | 0.52 | 0.09 | 6.68x10^-9^ | rs541663547 | 0.0006 |
| 7:127696479 | *OPN1SW* | 0.77 | 0.16 | 7.98x10^-7^ | NA | NA |
| 7:127953296 | *OPN1SW* | 0.38 | 0.08 | 1.66x10^-6^ | rs142674151 | 0.0022 |
| 7:128494922 | *OPN1SW* | 0.44 | 0.09 | 1.41x10^-6^ | rs201672146 | 0.0019 |
| 7:128499675 | *OPN1SW* | 1.56 | 0.14 | 1.04x10^-27^ | rs1187101372 | NA |
| 7:43489734 | *POLD2* | 1.38 | 0.25 | 5.54x10^-8^ | rs1035959449 | NA |
| 7:44646209 | *POLD2* | 0.95 | 0.18 | 1.23x10^-7^ | NA | NA |
| 2:24583125 | *POMC* | 1.85 | 0.35 | 8.95x10^-8^ | rs879385861 | NA |
| 6:34462967 | *RPL10A* | -1.91 | 0.22 | 5.50x10^-18^ | rs530446562 | 0.0008 |
| 6:35436943 | *RPL10A* | -0.77 | 0.13 | 7.99x10^-9^ | rs564411573 | 0.0008 |
| 6:35543534 | *RPL10A* | -1.91 | 0.22 | 5.50x10^-18^ | rs567496920 | 0.0002 |
| 12:11802124 | *TAS2R9* | 0.48 | 0.05 | 7.77x10^-23^ | rs932221793 | NA |
| 22:36678827 | *TMPRSS6* | 0.1 | 0.02 | 8.40x10^-11^ | rs371476289 | 0.00002 |
| 22:36745146 | *TMPRSS6* | 0.08 | 0.01 | 7.53x10^-10^ | rs147122501 | 0.0053 |
| 22:37823469 | *TMPRSS6* | 0.24 | 0.02 | 1.91x10^-34^ | NA | NA |
| 22:38093050 | *TMPRSS6* | 0.28 | 0.02 | 1.68x10^-52^ | rs1003630378 | NA |
| 22:38206144 | *TMPRSS6* | 0.28 | 0.02 | 1.68x10^-52^ | rs148188156 | 0.00078 |
| 22:38340452 | *TMPRSS6* | 0.28 | 0.02 | 1.68x10^-52^ | rs141057801 | 0.0007 |
| 22:38412776 | *TMPRSS6* | 0.17 | 0.02 | 5.99x10^-17^ | rs1025808525 | NA |
| 1:26485547 | *TRIM63* | 1.36 | 0.17 | 3.80x10^-15^ | rs530773694 | 0.0002 |
| 1:26560692 | *TRIM63* | 1.36 | 0.17 | 3.80x10^-15^ | rs961307336 | NA |
| 1:26869683 | *TRIM63* | 1.42 | 0.17 | 1.51x10^-16^ | rs188219941 | 0.00037 |
| 1:26872452 | *TRIM63* | 1.36 | 0.17 | 3.80x10^-15^ | rs201699710 | 0.0007 |
| 1:27028031 | *TRIM63* | 1.42 | 0.17 | 1.51x10^-16^ | rs759388842 | NA |
| 14:73943427 | *VRTN* | 0.17 | 0.01 | 1.88x10^-35^ | rs573822579 | 0.001 |
| 14:75686993 | *VRTN* | 0.11 | 0.01 | 1.30x10^-12^ | rs1305430246 | NA |
| 14:75707465 | *VRTN* | -0.11 | 0.01 | 1.30x10^-12^ | rs4903299 | 0.0076 |
| 19:19120659 | *ZNF101* | 3.53 | 0.21 | 3.96x10^-61^ | NA | NA |
| 19:19529415 | *ZNF101* | 2.07 | 0.25 | 2.13x10^-16^ | rs1405001784 | NA |
| 19:19120659 | *ZNF253* | 3 | 0.24 | 7.47x10^-36^ | NA | NA |
| 19:19529415 | *ZNF253* | 1.67 | 0.27 | 3.43x10^-10^ | rs1405001784 | NA |

NA – not available

* Map position according to GRCh37 assembly

**Supplementary Table 5**. eGene targets of both rare and common eQTLs in blood and brain

| **Blood** | | | | | **Brain** | | | | | | | | | |  |
| --- | --- | --- | --- | --- | --- | --- | --- | --- | --- | --- | --- | --- | --- | --- | --- |
| ABCA7 | DPPA4 | LRRC6 | RNF181 | | |  | | | ACOT1 | | | | |  |  |
| ABHD2 | DSC2 | MAF1 | RPA2 | | |  | | | ADAL | | | | |  |  |
| ACOX1 | EBPL | MAPKAPK3 | RPS23 | | |  | | | ANKK1 | | | | |  |  |
| ACSL6 | ECHDC3 | MEGF9 | RSRC1 | | |  | | | ANKRD30BL | | | | |  |  |
| ACSM1 | ENGASE | MGAM | S100A12 | | |  | | | ANXA9 | | | | |  |  |
| ADAMTSL4 | ENTPD1 | MMP24 | S100P | | |  | | | C10orf107 | | | | |  |  |
| AGPAT1 | EPB41L4A | MMP9 | SHC1 | | |  | | | C2orf74 | | | | |  |  |
| AIG1 | ESPN | MPC2 | SIGLEC5 | | |  | | | C5orf17 | | | | |  |  |
| ALOX5AP | EXOC2 | MRPL10 | SIGLEC9 | | |  | | | CCDC163P | | | | |  |  |
| APRT | EXOC4 | MRPS7 | SIN3B | | |  | | | CCDC173 | | | | |  |  |
| ARID3B | FAAH | MRVI1 | SLC22A1 | | |  | | | CYP2D6 | | | | |  |  |
| ARL17A | FCGBP | MS4A6A | SLPI | | |  | | | DNLZ | | | | |  |  |
| ARRB2 | FCGR2B | MXI1 | SMAD1 | | |  | | | FAM154B | | | | |  |  |
| ARSG | FKBP1A | MYOM1 | SMAP1 | | |  | | | GNMT | | | | |  |  |
| ASAH1 | FNBP1L | MZT2A | SNX19 | | |  | | | GUF1 | | | | |  |  |
| ASPRV1 | FOLR3 | NLRP1 | SPATA20 | | |  | | | HLA-A | | | | |  |  |
| ATG7 | FST | NLRP3 | SPPL3 | | |  | | | HLA-DOB | | | | |  |  |
| ATL1 | GAD1 | NMRAL1 | SSH3 | | |  | | | HLA-DRB1 | | | | |  |  |
| ATP6V0D1 | GCAT | NSMAF | ST6GALNAC2 | | |  | | | HLA-DRB5 | | | | |  |  |
| ATP6V1D | GSTA3 | NUDT2 | STYXL1 | | |  | | | ***HP*** | | | | |  |  |
| ATXN7L3B | GSTA4 | NUMA1 | TAC3 | | |  | | | HPR | | | | |  |  |
| BEGAIN | GTF3C3 | NUP107 | TAP2 | | |  | | | IL27 | | | | |  |  |
| CARS2 | HAL | NUP50 | TIAM2 | | |  | | | ***LDHC*** | | | | |  |  |
| CCDC122 | HAUS4 | PADI2 | TMCC3 | | |  | | | LYPD8 | | | | |  |  |
| CCDC146 | HEATR6 | PADI4 | TMED6 | | |  | | | MMRN1 | | | | |  |  |
| CD300A | HEBP1 | PBX2 | TMEM163 | | |  | | | NBPF16 | | | | |  |  |
| CD300C | HEBP2 | PCYT1A | TMEM51 | | |  | | | NPIPA2 | | | | |  |  |
| CD36 | HIP1 | PDLIM5 | TOP1MT | | |  | | | NPIPA8 | | | | |  |  |
| CDA | HMBOX1 | PEX6 | TP53I3 | | |  | | | OPN1SW | | | | |  |  |
| CDK5R1 | HOXB2 | PGM1 | TREML4 | | |  | | | POMC | | | | |  |  |
| CFL1 | HOXB3 | PGM2L1 | TSPAN16 | | |  | | | PSORS1C1 | | | | |  |  |
| CHI3L1 | ***HP*** | PGM5 | TUBB | | |  | | | ***RBPMS2*** | | | | |  |  |
| CHURC1 | HP1BP3 | PISD | TUBB2A | | |  | | | RNF39 | | | | |  |  |
| CISD1 | HSD17B13 | PLA2G4C | TUBB6 | | |  | | | RPRD2 | | | | |  |  |
| CISD2 | HSPA1B | PLAGL1 | UBE4B | | |  | | | TRIM63 | | | | |  |  |
| CLN6 | IER3 | PNKD | UFSP2 | | |  | | | YBEY | | | | |  |  |
| CLTCL1 | IKZF3 | PNLDC1 | UHRF1BP1 | | |  | | | ZNF253 | | | | |  |  |
| CLYBL | IMPA2 | POLR1A | ULK4 | | |  | | | ZNF514 | | | | |  |  |
| CPA5 | INPP1 | PPP3CA | UQCRC1 | | |  | | | ZNRD1 | | | | |  |  |
| CPVL | IP6K2 | PPT1 | USP10 | | |  | | | ZSCAN31 | | | | |  |  |
| CREB5 | KIAA1191 | PYGB | UTS2 | | |  | | |  | | |  |  |  |  |
| CTNNAL1 | KIF1B | RAB27A | UVSSA | | |  | | |  | | |  |  |  |  |
| CTSK | ***LDHC*** | RABEP1 | VNN2 | | |  | | |  | | |  |  |  |  |
| CTSW | LGALS4 | RALBP1 | WARS2 | | |  | | |  | | |  |  |  |  |
| CXCL1 | LGALS8 | RBM23 | XRRA1 | | |  | | |  | | |  |  |  |  |
| DAD1 | LILRA1 | RBMS1 | YTHDC2 | | |  | | |  | | |  |  |  |  |
| DEF6 | LILRA2 | RBP7 | ZDHHC2 | | |  | | |  | | |  |  |  |  |
| DGCR8 | LIN7A | ***RBPMS2*** | ZNF502 | | |  | | |  | | |  |  |  |  |
| DHRS4 | LPXN | RHD | ZNF605 | | |  | | |  | | |  |  |  |  |
| DNAJC15 | LRRC2 | RIN1 | ZP3 | | |  | | |  | | |  |  |  |  |
| DOK4 | LRRC4 | RNF14 |  |  | | | |  | | |  |  |  |  |  |
| eQTLs common to blood and brain are highlighted in ***bold italics*** | | | | | | |  | | |  | | |  | | |
